# Perfluoroalkyl acid precursor or weakly fluorinated organic compound? A proof of concept for oxidative fractionation of PFAS and organofluorines

**DOI:** 10.1007/s00216-024-05590-5

**Published:** 2024-10-12

**Authors:** Jonathan Zweigle, Apollonia Schmidt, Boris Bugsel, Christian Vogel, Fabian Simon, Christian Zwiener

**Affiliations:** 1 Department of Geosciences, Environmental Analytical Chemistry, University of Tübingen, Schnarrenbergstraße 94-96, 72076 Tübingen, Germany; 2 Division 1.1 – Inorganic Trace Analysis, Federal Institute for Materials Research and Testing, Richard-Willstätter-Straße 11, 12489 Berlin, Germany; 3 Division 4.3 – Contaminant Transfer and Environmental Technologies, Federal Institute for Materials Research and Testing, Unter Den Eichen 87, 12205 Berlin, Germany

**Keywords:** Organofluorine mass balance, PFAS, Organofluorines, PhotoTOP, PFAA-precursors, TFA-precursors

## Abstract

Organofluorine mass balance approaches are increasingly applied to investigate the occurrence of per- and polyfluoroalkyl substances (PFAS) and other organofluorines in environmental samples more comprehensively. Usually, complex samples prevent the identification and quantification of every fluorine-containing molecule. Consequently, large unidentified fractions between fluorine sum parameters such as extractable organic fluorine (EOF) and the sum of quantified analytes are frequently reported. We propose using oxidative conversion to separate (unidentified) weakly fluorinated compounds (e.g., pesticides, pharmaceuticals) from PFAA-precursors (perfluoroalkyl chain lengths ≥ C_6_). We show with three organofluorine model substances (flufenamic acid, diflufenican, pantoprazole) that CF_3_-groups or aromatic fluorine can be quantitatively converted to inorganic fluoride and trifluoroacetic acid (TFA) by applying PhotoTOP oxidation (UV/TiO_2_). The principle of fluorine separation in mixtures is demonstrated by the oxidation of the three weakly fluorinated compounds together with the PFAA-precursor 6:2/6:2 fluorotelomer mercaptoalkyl phosphate diester (FTMAP). After oxidation, the products F^−^ and TFA were separated from PFCAs (> C_4_) by SPE, and the fractions were analyzed individually. Closed mass balances both with and without the addition of organic matrix were achieved. Eventually, the fluorine balance was verified by total fluorine measurements with combustion ion chromatography (CIC). The proposed methods should be considered a proof of concept to potentially explain unidentified fractions of the EOF, especially if compounds with low fluorine content such as pesticides, pharmaceuticals, and their transformation products contribute largely to the EOF. Future studies are needed to show the applicability to the complexity of environmental samples.

**Graphical Abstract:**

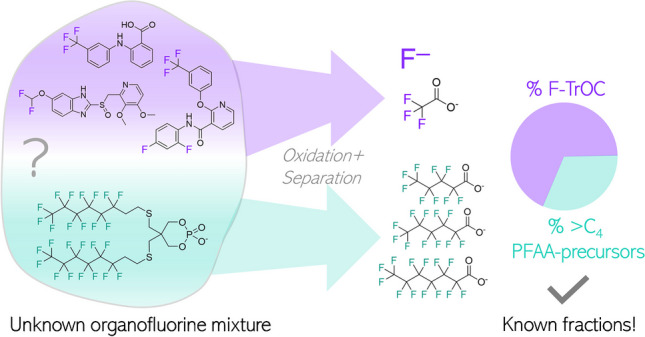

**Supplementary Information:**

The online version contains supplementary material available at 10.1007/s00216-024-05590-5.

## Introduction

Per- and polyfluoroalkyl substances (PFAS) gained increasing attention in recent years due to their widespread use and detection all over the world [1, 2]. Both the high persistence and adverse health effects of selected PFAS on humans and the environment are of particular concern [3, 4]. Recent actions in the European Union aim to restrict PFAS as an entire chemical class with immense impact on many industrial branches [5]. This includes a huge number of chemicals since the updated OECD definition now considers all chemicals comprising a CF_3_- or a -CF_2_-groups (without H or other halogens) as PFAS which involves also many pesticides and pharmaceuticals (partially excluded from planned regulations) [6–8]. A comprehensive characterization of PFAS in the environment is challenging due to the large number of potential compounds to consider paired with the limited availability of authentic reference standards [9, 10]. Besides conventional target screening and non-target screening (NTS) approaches that consider the identity of individual PFAS, fluorine mass balance approaches, in particular extractable organic fluorine (EOF), have become more relevant in recent years [11, 12]. Numerous studies all over the world report high fractions of unidentified organofluorine in various matrices when comparing the quantity of individual compounds with the EOF [13–18]. This highlights the need for novel approaches to explain the type or class of organofluorines from which this unknown part arises. Depending on the sample complexity, the identification of every single organofluorine is usually not possible especially since the variety in environmental samples can be rather high [19]. Especially weakly fluorinated compounds, in the following referred to as fluorinated trace organic compounds (F-TrOCs), such as pesticides, pharmaceuticals, and their transformation products (TPs) can be difficult to identify during suspect- and NTS since typical PFAS prioritization approaches often cannot prioritize them (e.g., mass defect, m/C ratio, or collision cross-section, no homologues series) [20]. Furthermore, in the case of TPs, often only semi-quantification is possible. Different studies put strong effort into the identification of the unknown organofluorine fractions, which, however, could only be explained in a few samples [21, 22]. Since a comprehensive identification and quantification of each organofluorine compound is not possible in complex samples, approaches based on chemical conversion of precursors to their terminal products (e.g., perfluoroalkyl acids (PFAAs)) such as the total oxidizable precursor (TOP) assay and modified versions were developed [23–25]. Oxidation approaches can be classified in between the analysis of single compounds and sum parameters (e.g., EOF) since they can cover a wide range of compounds while partially conserving structural and quantitative information (depending on the sample matrix). To date, oxidative conversion approaches were mainly applied to characterize PFAA-precursors (e.g., at least 4 perfluorinated carbon atoms); however, it was not investigated whether also weakly fluorinated F-TrOCs (e.g., compounds with single F, aromatic F, or CF_3_-groups) can also be included. Since for many environmental samples such as sewage sludge [21] and suspended particulate matter (SPM) [26] it was shown that the fractions of F-TrOCs (e.g., pharmaceuticals) in the EOF can be higher than “conventional” PFAS (e.g., chain length ≥ C_4_), it would be of high relevance to separate those two fractions. Therefore, the present study aims to develop a method to convert organofluorine mixtures (both weakly fluorinated F-TrOCs and PFAA-precursors) via PhotoTOP oxidation (UV/TiO_2_) to PFAAs and fluoride and subsequently separate them by solid phase extraction (SPE) to gain more specific information about the type of organofluorine present in complex samples [24]. The PhotoTOP was chosen since unlike the conventional TOP assay it mostly conserves the perfluoroalkyl chain lengths of precursors (as (n-1); e.g., PFHxA from a 6:2 telomer-based precursor, no formation of ultra-short chain PFCAs), allowing a clearer distinction between PFAA-precursors with chain lengths ≥ C_6_ and weakly fluorinated compounds (e.g., fluoropesticides) [27]. The TOP assay usually results in a stronger chain shortening yielding also small fractions of trifluoroacetic acid (TFA) from a 6:2 telomer-based precursor making a distinction between a TFA-precursor and a longer chain PFAA-precursor more difficult. Furthermore, the absence of salts in the PhotoTOP simplifies the sample preparation and subsequent analysis.

**Fig. 1:**
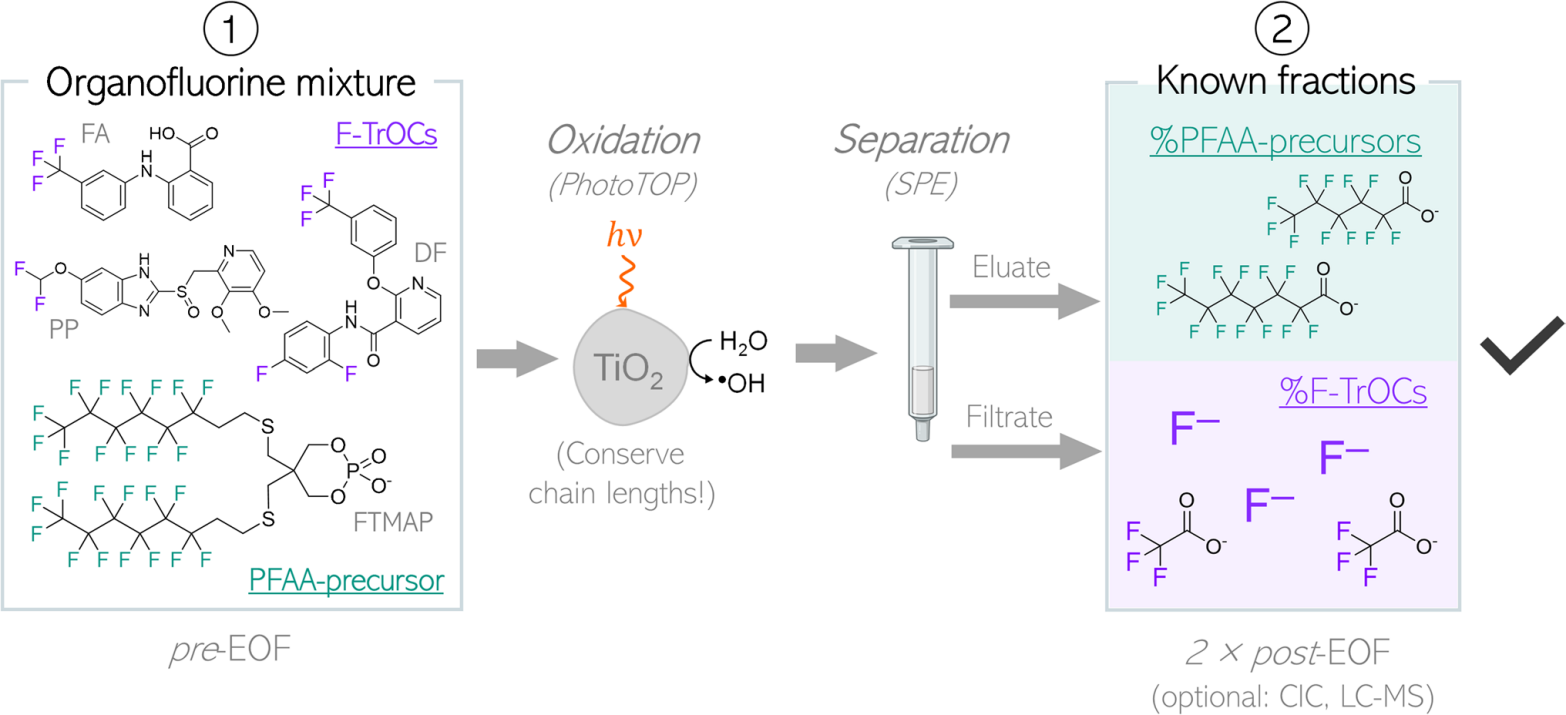
Schematic illustration of the proposed approach to separate the EOF fractions from fluorinated trace organic compounds (F-TrOCs) from PFAA-precursors with perfluoroalkyl chain lengths > C_4_. The separated fractions can both be analyzed by EOF or if available for PFAAs, TFA, and fluoride with suitable methods. Abbreviations of the used compounds in this study: FA, flufenamic acid; DF, diflufenican; PP, pantoprazole

When looking at the FLUOROPEST (318 compounds) and FLUOROPHARMA (290 compounds) suspect lists from the PFAS PubChem Tree, ~ 33% and ~ 49% of each list have at least one aromatic fluorine atom, and ~ 60% and ~ 28% have at least one CF_3_-group, respectively (downloaded from the PubChem PFAS Tree) [28]. To first investigate the oxidation behavior of weakly fluorinated F-TrOCs, three model substances (flufenamic acid (FA), CAS-Nr: 530–78-9; diflufenican (DF), CAS-Nr: 83,164–33-4, pantoprazole (PP), CAS-Nr: 102,625–70-7) were selected that cover typical fluorine functional groups such as perfluoro CF_3_-, aromatic F, and non-perfluoro groups (CF_2_H-group, non-PFAS) (for structures, see Fig. 1). FA and PP are pharmaceuticals, while DF is an herbicide. All three compounds are commonly detected in the environment [29–31]. They were oxidized individually to demonstrate their quantitative conversion to stable end products (TFA and fluoride (F^−^)). Mixtures of the weakly fluorinated F-TrOCs and a 6:2 telomer-based PFAA-precursor (6:2 fluorotelomer mercaptoalkyl phosphate diester; FTMAP) were oxidized together and the oxidation products were separated into two fractions by SPE and analyzed individually. Eventually, the fluorine balance was further confirmed by total fluorine measurements by combustion ion chromatography (CIC). The advantages and limitations of the proposed method are highlighted.

## Materials and methods

### Chemicals and reagents

LC/MS grade water, methanol (MeOH), and ammonium acetate (NH_4_Ac) were purchased from Thermo Fisher Scientific. Titanium(IV) dioxide (TiO_2_ anatase, powder, 99.8% trace metal basis), FA, PP-sodium, and a humic acid standard (CAS: 1415–93-6) were ordered from Sigma-Aldrich. DF was purchased from Dr. Ehrenstorfer, TFA from Alfa Aesar, and NaF from Merck. The 6:2/6:2 fluorotelomer mercaptoalkyl phosphate diester (FTMAP) was synthesized in-house (for details, see [32]). PFCA reference standards (PFBA, PFPeA, PFHxA, PFHpA, PFOA, PFNA, PFDA) were ordered from Wellington Laboratories (PFAC-MXC). To prepare aqueous solutions with different DOC contents, 10 mg of humic acid standard was mixed with 250 mL of LC–MS grade water and ultrasonicated for 15 min followed by a filtration step. From the resulting solution, different dilutions were prepared and measured for DOC with a TOC analyzer (Elementar HighTOC).

### PhotoTOP oxidation

The PhotoTOP oxidation experiments were performed identically as described in the original paper [24]. Twenty milligrams of TiO_2_ particles was weighed in 20-mL EPA glass vials and coated with the respective standards or sample extracts (in MeOH) which were evaporated at room temperature overnight. For single experiments with the F-TrOCs (DF, FA, and PP), 10 µM of each analyte was spiked while for the mixture experiments, 0.2 µmol F of each F-TrOC and 0.6 µmol F from the PFAA-precursor (6:2/6:2 FTMAP) were combined. In separate experiments, 0.05 µmol of each F-TrOC and 0.15 µmol 6:2 FTMAP were used in the presence of three DOC concentrations (~ 0.2, ~ 1, and ~ 5 mg/L aqueous solutions made from a humic acid mixture, details in the SI). After evaporation of the MeOH, 23 mL of water and magnetic stirrers were added followed by 5 min of ultrasonication. Oxidation under UV irradiation was 6 h. Depending on the experiment, 0.4-mL samples were taken at certain time intervals and mixed with 0.4 mL of MeOH followed by 10-min centrifugation at 20,817 relative centrifugal forces (rcf). For fluorine mass balance calculations, in every experiment, the initial moles of fluorine were quantified in a freshly and likewise prepared MeOH solution. This was necessary, since for the very hydrophobic FTMAP, the initial concentration on the TiO_2_ particles cannot be quantified accurately [24]. The fluorine balance was calculated on a molar basis according to Eq. 1:

1 $$\%\mathrm F=\frac{\mathrm{F}_{\mathrm{C}_4-{\mathrm C}_{10}\;\mathrm{PFCA}s}+\mathrm{F}_{\mathrm{TFA}}+\mathrm F^-}{\sum_{}\mathrm{F}_{\mathrm{Precursors}}}\cdot\;100\%\\$$

where F corresponds to the moles of fluorine in the form of PFCAs, TFA, fluorine, and all used precursors, respectively.

### Fluorine separation by SPE

To separate the oxidation products F^−^ and TFA from PFCAs (> C_4_), solid phase extraction (SPE) was used. OASIS HLB cartridges were chosen since the sorbent material is suitable for separating ultra-short chain PFAAs and fluoride from > C_4_ PFAAs. The cartridges (60 mg sorbent) were preconditioned with 1 mL MeOH followed by 1 mL water and a drying step. The remaining water from PhotoTOP oxidation (~ 20 mL, after removing the TiO_2_ via centrifugation) was passed through the cartridges (filtrate) and collected together with a subsequent washing step with 1 mL water with 3% MeOH (v/v). This filtrate fraction was further used for F^−^ and TFA analyses. The remaining PFCAs were eluted with 2 mL MeOH. As a quality control, tests with a PFCA standard mix demonstrated good recoveries for PFPeA and longer chain PFCAs while ~ 80% of PFBA was found in the filtrate (Figure S1).

### Instrumental analysis

DF, FA, PP, and C_4_-C_10_ PFCAs were quantified using an Agilent 1260 Infinity HPLC (Agilent Poroshell EC-C_18_ column; 2.1 × 100 mm, 2.7 µm particles at 40 °C) coupled to an Agilent 6550 quadrupole time-of-flight mass spectrometer (QTOF-MS). A 23-min gradient elution with solvents A: 95/5 water/MeOH + 2 mM ammonium acetate (NH_4_Ac) and B: 5/95 water/MeOH + 2 mM NH_4_Ac was applied with a flow rate of 0.3 mL/min (Table S1, Table S2 for instrument and source parameters). The injection volume was 5 µL and ionization was performed in positive electrospray ionization (ESI^+^) for DF, FA, and PP, and ESI^−^ for PFCAs and TFA.

TFA was measured using an isocratic method with a solvent ratio of A/B of 60/40. TFA was barely retained on the C_18_-column; however, quantification in the matrix-free PhotoTOP oxidation solution was possible (Figure S2). TFA was quantified using external calibration. All substances were verified by accurate mass and retention time (RT) and quantified with external calibration standards measured before and after the samples. Examples of extracted ion chromatograms (EICs) are shown in Figure S3. As quality control, after every 10th sample, MeOH blanks and a PFAS standard mixture were measured to monitor background signals and instrument response. No instrument drift or interfering blank signals of any of the investigated analytes were observed. After every injection, a needle wash was performed.

Fluoride (F^−^) was quantified with ion chromatography (IC) using a Metrohm 930 Compact IC Flex with Milli-Q water with a conductivity < 0.05 µS/cm^2^. Anions were separated on a Metrosep A Supp 5 column (150 × 4.0 mm, 5 µm particles) using an eluent with 3.2 mM Na_2_CO_3_ and 1 mM NaHCO_3_ at a flow rate of 0.7 mL/min with a Metrosep A Supp 5 Guard 4.0 pre-column and a suppressor. One or 8 mL of filtered samples was injected. The quantification range was 20–2000 µg/L F^−^. As quality control, all samples were measured in their original form and in a dilution of 1:10, and blanks were included in every measurement batch which showed F^−^  < LOD.

For TF analysis, the MeOH or water extracts were directly combusted and subsequently analyzed via CIC (combination of ion chromatography (IC; ICS Integrion, Thermo Fisher Scientific) and a combustion and absorption unit (AQF-2100H, GA-210, Mitsubishi Chemical Analytech)) [33]. Aliquots of all samples (250 µL) were injected automatically into a quartz wool–filled ceramic boat prior to measurement by CIC. The combustion unit consisted of an autosampler (ASC-270LS) connected to the induction furnace (AQF-2100H) operating between 1000 and 1050 °C under a flow of O_2_ (300 mL/min) and Ar (150 mL/min). Combustion gases were absorbed in a freshly prepared 3 mM NH_3_ solution, added with an internal standard for monitoring the exact absorption volume by IC. For all measurements, the water supply level was set to “2,” combined with the small absorption volume (~ 11 mL). A 100-µL aliquot was injected into the ion chromatography using Dionex IonPac AG30 (2 × 50 mm) as guard column and Dionex IonPac AS30 (2 × 250 mm) as analytic column, both maintained at a constant column temperature of 30 °C. Chromatographic separation was directed by an automated KOH eluent generator, controlled by an optimized gradient program (5.5 to 80 mM) at a constant flow rate of 0.38 mL/min. Fluoride ions were analyzed by a conductivity detector using 50 mM H_2_SO_4_ as suppressor regenerant. For calculation of detected peak areas and fluoride concentrations, chromatography data system Chromeleon 7.2.10 (Thermo Fisher Scientific) was used. Calibration standards were made from a NH_4_F stock solution. A ten-point calibration curve with concentrations from 48 to 1728 µg/L F^−^ was recorded.

## Results and discussion

### Oxidation of individual organofluorines (F-TrOCs)

**Fig. 2:**
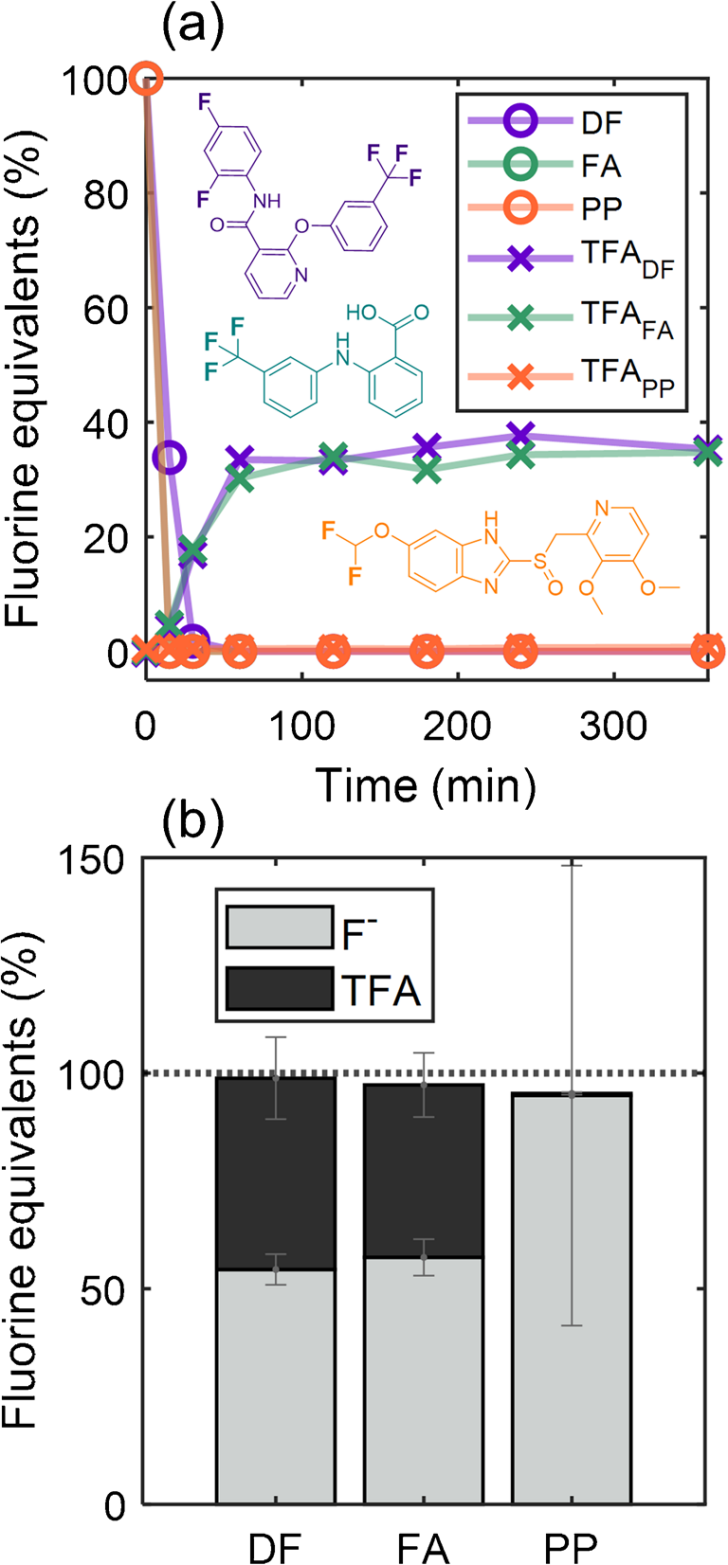
**a** Individual PhotoTOP oxidation experiments of the F-TrOCs diflufenican (DF), flufenamic acid (FA), and pantoprazole (PP) over the course of 6 h. After a rapid degradation of DF and FA, TFA formation was observed, while PP expectedly did not yield TFA due to the absence of a CF_3_-group (see Fig. 1). **b** Fluorine mass balance after 6 h of oxidation from duplicate experiments. Error bars correspond to the standard deviation

To test the hypothesis that weakly fluorinated compounds (F-TrOCs) can be quantitatively converted to TFA and other stable end products via the PhotoTOP, three model compounds (DF, FA, and PP; see Fig. 1) were selected with common fluorine functionalities (CF_3_-group, aromatic F, and other non-perfluorinated F substituents) found in many pesticides and pharmaceuticals. First, individual PhotoTOP oxidation of the three F-TrOCs was investigated (Fig. 2a). All three F-TrOCs showed rapid degradation after approximately 30 min. For FA and DF, the formation of TFA was observed while in the case of PP, no TFA was detected. This was expected since PP has a R-O-CF_2_H group which cannot be transformed to TFA. After ~ 2 h of oxidation, production of TFA from FA and DF reached a plateau, indicating a complete reaction. Although DF has two aromatic fluorine atoms in addition to the CF_3_-group, approximately 35% (on a molar fluorine basis) of both DF and FA were transformed to TFA, showing that a larger fraction of the fluorine in the CF_3_-group of FA was transformed to other products. The plateau shows that once formed, TFA is stable in the presence of OH-radicals generated in the PhotoTOP. Due to the higher sample volume required for F^−^ analysis (flushing of the sample loop), F^−^ was measured only after 6 h of PhotoTOP oxidation and no time trend could be acquired. A fluorine balance on a molar basis was then calculated according to Eq. 1. The fluorine balance shows that the remaining fraction of fluorine of the F-TrOCs was mineralized to F^−^ (Fig. 2b). For DF, FA, and PP, the sum of TFA and F^−^ revealed a closed fluorine balance of 99% ± 13%, 97% ± 12%, and 95% ± 54%, respectively (*n* = 2). Interestingly, the TFA/F^−^ ratio of DF and FA was very similar although DF has two additional aromatic F-atoms, showing that different fractions of the CF_3_-groups were converted to TFA. This shows that the TFA yield (and mineralization to F^−^) from CF_3_-groups can be different, depending on their location (and generally dependent on the chemical structure) and that a direct conclusion on the structure of the precursor is not possible from TFA formation. In the case of PP, the variance between two separate experiments was higher, with fluorine balances of 133% and 57%. A more sensitive IC method would be beneficial and could decrease the high variance. In general, the results demonstrate that it is possible to quantitatively convert F-TrOCs to TFA and F^−^ via PhotoTOP oxidation. While TFA-formation can be an indication of CF_3_-groups, yields can be different for structurally different compounds. However, if fluoride and TFA are the main TPs from F-TrOC, the different ratios of TFA and fluoride yields should not change the fluorine mass balance since the fluorine from both TPs is combined.

### Oxidation of mixtures of short (F-TrOCs)- and long-chain PFAA-precursor

**Fig. 3:**
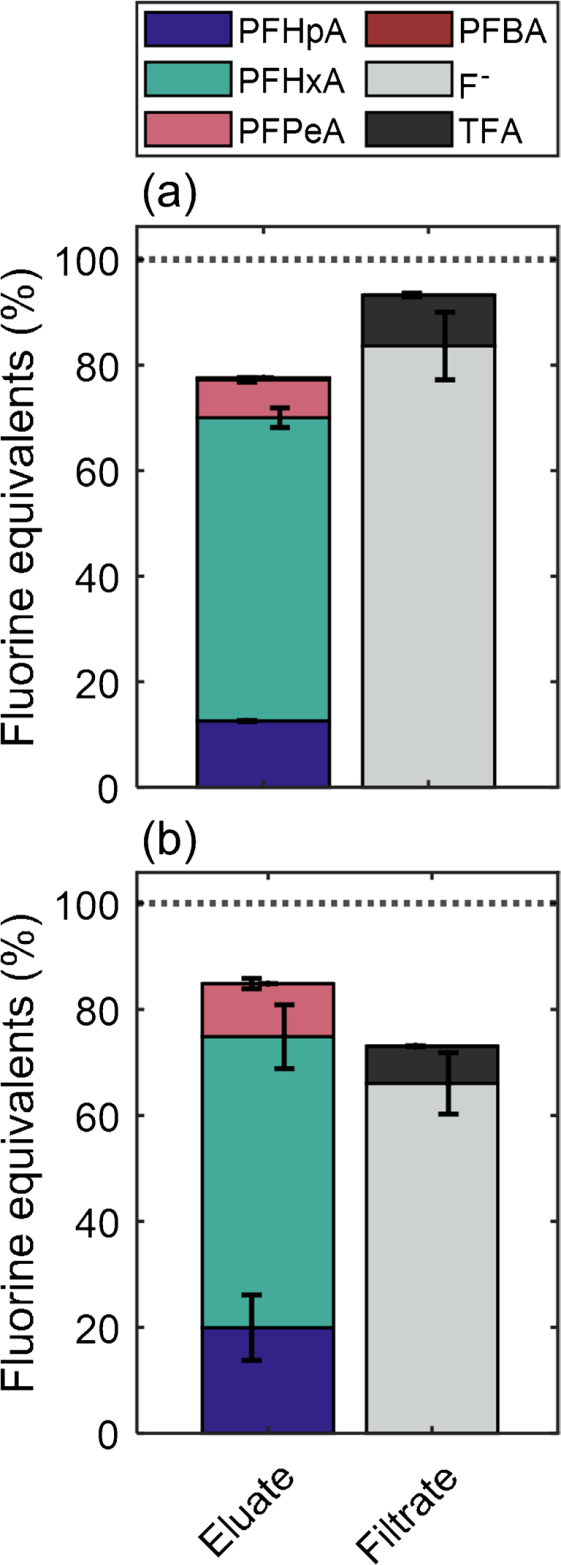
Molar fluorine balances of PhotoTOP oxidation experiments of mixtures of DF, FA, PP, and 6:2 FTMAP. It becomes obvious that the oxidation products (> C_4_ PFAAs) of FTMAP can be recovered in the eluate, while the fluorine from the F-TrOCs can be found in the filtrate (TFA and F^−^). The moles of fluorine of the F-TrOCs were identical to 6:2 FTMAP [*n*_F_(DF + FA + PP) = *n*_F_(FTMAP)]. **a** Two oxidation experiments without matrix (1.2 µmol F). **b** Three experiments with ~ 0.2, ~ 1, and ~ 5 mg/L DOC (dissolved humic acid standard, 0.3 µmol F). Error bars correspond to the standard deviation of duplicates. The PFCAs from the eluent correspond the PFAA-precursor (6:2 FTMAP) and the filtrate to the three F-TrOCs

In the next step, mixtures of the three F-TrOCs (DF, FA, PP) and 6:2 FTMAP were oxidized in the PhotoTOP. The goal of this experiment is to prove the hypothesis that the fluorine in F-TrOCs can be separated from the fluorine in longer chain PFAA-precursors (here C_6_) via oxidation and subsequent SPE separation (F^−^  + TFA vs. > C_4_-PFCAs). The PhotoTOP has the advantage that the chain length of precursors is mostly conserved after oxidation allowing a rather accurate determination of perfluoroalkyl chain length. For instance, a 6:2 telomer-based precursor is transformed to mainly PFHxA, PFHpA, and a much smaller fraction of PFPeA, while ultra-short chain PFCAs are negligible [24]. To test the hypothesis of oxidative fractionation, the identical molar quantity of fluorine in form of the three F-TrOCs (0.2 µmol F each, total 0.6 µmol) and 6:2 FTMAP (0.6 µmol F) was oxidized for 6 h. The remaining aqueous solution was subjected to SPE where both filtrate (sample through cartridge) and eluate were collected and individually analyzed (see also Fig. 1). The SPE method was shown to discriminate ultra-short chain PFCAs up to C_4_ from PFCAs > C_4_. After oxidation, in the eluate, PFPeA, PFHxA, and PFHpA added up to a fluorine balance of 78% ± 2% of the initially spiked 6:2 FTMAP, showing that an almost quantitative conversion was achieved (Fig. 3a). Since no PFBA was detected (similar to PhotoTOP oxidation of other precursors), no TFA-formation is expected from 6:2 FTMAP [24]. The chain length distribution from 6:2 FTMAP (ratios of PFPeA, PFHxA, and PFHpA) is in good accordance with previous PhotoTOP oxidation experiments with other 6:2 telomer-based precursors [24]. It should be noted that the slight chain shortening (e.g., PFHxA is shortened by one CF_2_-unit compared to the original fluorinated alkyl chain of 6:2 FTMAP) results in a slight production of F^−^ which is not included in the fluorine balance. Since the original chain length of the precursor is known in this experiment, the theoretical inclusion of this produced F^−^ leads to a fluorine balance of 92% (closed mass balance). 93% ± 7% of the fluorine from the three F-TrOCs could be recovered in the filtrate in form of TFA and F^−^ as well (Fig. 3a). Interestingly, most of the F-TrOCs were mineralized to F^−^ and only ~ 12% of the fluorine was recovered as TFA. Overall, the results demonstrate that > C_4_-PFAA-precursors can be discriminated from organofluorines with CF_3_-groups and aromatic F by this approach (> C_4_ PFCAs vs. TFA + F^−^).

To further consider the presence of matrix which is one of the most challenging difficulties of oxidation techniques such as the TOP assay or PhotoTOP [27, 34, 35], three oxidation experiments with different dilutions of an aqueous extract of a commercial humic acid standard were performed. The extracts had ~ 0.2, ~ 1, and ~ 5 mg/L DOC and only one-quarter of each analyte was used (0.3 µmol F in total). In the pooled experiments with DOC content, 85% ± 13% of the fluorine from 6:2 FTMAP and 73% ± 6% of the fluorine from the three F-TrOCs could be recovered after oxidation and SPE in the eluate and filtrate, respectively. This demonstrates that also in the presence of organic matrix a quantitative conversion and separate detection of the fluorine from the two different kinds of fluorinated compounds might be possible. Due to the photocatalytic nature of UV/TiO_2_ oxidation, samples with high DOC content can also be oxidized for a longer time to ensure the likelihood of a complete oxidation of organofluorines and PFAA-precursors.

### Fluorine balance confirmation by CIC

**Fig. 4:**
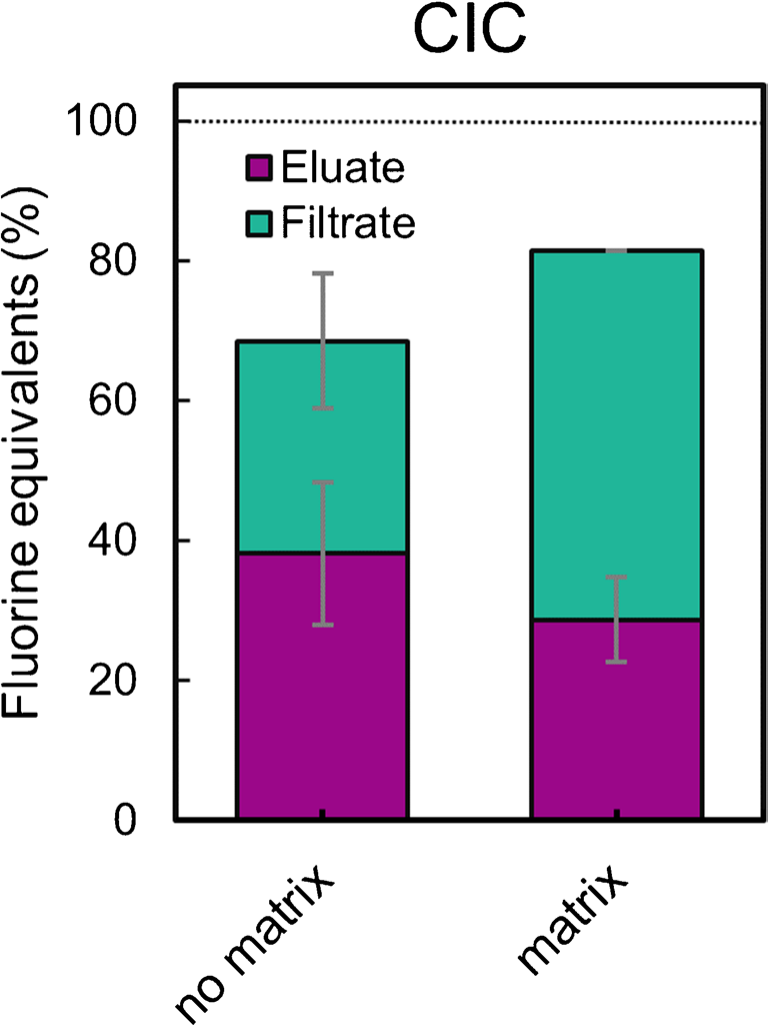
Molar fluorine balances of PhotoTOP oxidation experiments of mixtures of DF, FA, PP, and 6:2 FTMAP measured by CIC (for comparison with quantification of individual analytes, see Fig. 3). Here, the fractions of F-TrOCs (filtrate) and the PFAA-precursor FTMAP (eluate) are shown together

To verify the fluorine balances which are based on the quantification of single compounds (> C_4_ PFCAs, TFA, and F^−^), the same samples as described in the previous section were measured by CIC for total fluorine before oxidation and both filtrate and eluate after oxidation and SPE separation. The results confirmed that the separation in PFCAs > C_4_ in the eluate and TFA, F^−^, and other low molecular oxidation products in the filtrate also works for fluorine sum parameter measurements (Fig. 4). For the experiment without matrix, fluorine recoveries for the F-TrOC were 61% ± 19% and for 6:2 FTMAP 76% ± 20%, respectively. The addition of DOC resulted in fluorine balances of 105% (only one measurement) (F-TrOCs) and 57% ± 12% (6:2 FTMAP). The fluorine recoveries were slightly lower in the CIC measurements than the quantification of individual compounds. The direct comparison of the total fluorine balances (eluate + filtrate) after oxidation without matrix was 85% and 68% based on single compounds quantification and CIC, respectively. In the presence of matrix, the fluorine balances were 79% and 81%, respectively. Gaps in the fluorine balance could have arisen from sample preparation, from measurement accuracy, or from the formation of minor fractions of volatile oxidation products that were not assessed in this study. Given the fact that the unknown organofluorine fractions in environmental and human samples reach up to > 90%, and the uncertainties associated with oxidative methods such as the TOP assay are rather high, our method can be considered for future investigations of the origin of unknown organofluorines with the here achieved accuracies [17, 35, 36].

## Advantages and limitations of the proposed method

The proposed method demonstrates that a combination of oxidative conversion and SPE allows the separation of fluorine of unknown F-TrOCs from PFAA-precursors with perfluoroalkyl chain lengths ≥ C_6_ (e.g., 6:2 telomer-based compounds). This approach can help to partially elucidate the frequently reported unknown fraction in organofluorine mass balance approaches without the identification of all compounds. In environmental samples, the comparison of PFAAs, TFA, and fluoride in the original sample vs. after oxidation can be applied (similar as typically done for TOP assay approaches). In complex samples with high content of F-TrOCs (and their TPs), it is impossible to quantify each individual compound that we suggest being a potential application of our approach (e.g., sewage sludge [21]). Furthermore, the formation potential of both TFA- and PFAA-precursors in environmental samples can be assessed together.

The application to complex environmental samples has some limitations that need to be assessed in the future. Usually, fluorine contents are lower than in the experiments performed in the present study which requires low LOQs for EOF in sample extracts or water samples. Instead, after oxidation, more sensitive methods such as LC–MS/MS for PFCAs, IC for F^−^, and LC–MS/MS or IC-MS/MS for TFA could also be used without the SPE separation which is required mainly for fluorine sum parameter measurements (e.g., TF via CIC). Furthermore, it might be necessary to initially remove (or quantify) all inorganic F^−^ and TFA in environmental samples before applying the oxidation. With the conventional persulfate-based TOP assay, the proposed method is not possible due to chain shortening (e.g., up to 25% TFA yield from different precursors [34]) and high salt content that complicates the analysis of an SPE filtrate by IC (very high Cl^−^ concentrations after pH adjustment after oxidation).

In environmental samples, the organic matrix content can also complicate oxidation due to scavenging of OH-radicals which is a common problem in the TOP assay. Therefore, it is recommended to add an indicator such as isotopically labeled PFOSA to verify a quantitative oxidation [34]. With the PhotoTOP, the issue of high matrix content might be overcome by increasing oxidation times (photocatalytic OH-radical formation). Nevertheless, the influence of matrix on oxidation methods such as TOP and PhotoTOP needs further investigation.

Overall, the proposed method is a promising approach to gain relevant structural information on different types of unknown organofluorine in the environment, even with sum parameters such as the EOF (before and after oxidation and separation). The suggested method could further benefit from an improved SPE method to allow the separation of C_4_-precursors from F-TrOCs. Depending on the complexity of fluorinated compounds in environmental samples, a clear distinction between weakly fluorinated compounds and PFAA-precursors as shown here might not be possible and an overlap of TPs of both fractions after oxidation is likely. Nevertheless, the inclusion of TFA and fluoride into oxidation-based PFAS characterization could provide valuable information on the characteristics of PFAS contamination (weakly fluorinated pesticides or pharmaceuticals vs. PFAA-precursor with more than 4 perfluorinated carbon units) which is often not achievable by other methods. We encourage other researchers to further optimize, apply, and prove the presented approach in combination with environmental samples.

## Supplementary Information

Below is the link to the electronic supplementary material.

Supplementary file1 (PDF 917 KB)
