# Supplementary material for: Perfluoroalkyl acid precursor or weakly fluorinated organic compound? A proof of concept for oxidative fractionation of PFAS and organofluorines
Source: Anal Bioanal Chem. 2024 Oct 12;416(29):6799–808. doi: 10.1007/s00216-024-05590-5 (PMC11579176; doi:10.1007/s00216-024-05590-5)
Supplement: Supplementary file 1 — Supplementary file1 (PDF 917 KB) [file 216_2024_5590_MOESM1_ESM.pdf]

Supporting Information

Perfluoroalkyl acid precursor or weakly fluorinated organic compound? A proof of concept for oxidative fractionation of PFAS and organofluorines

Jonathan Zweigle,[+,*] Apollonia Schmidt,[+] Boris Bugsel,[+] Christian Vogel,[∇] Fabian Simon,[‖] Christian Zwiener[+]

[+]Environmental Analytical Chemistry, Department of Geosciences, University of Tübingen, Schnarrenbergstraße 94-96, 72076 Tübingen, Germany

[‖]Federal Institute for Materials Research and Testing (BAM), Division 1.1 – Inorganic Trace Analysis, Richard-Willstätter-Straße 11, 12489 Berlin, Germany

[∇]Federal Institute for Materials Research and Testing (BAM), Division 4.3 – Contaminant Transfer and Environmental Technologies, Unter den Eichen 87, 12205, Berlin, Germany

[*]*Corresponding author*

Contents

- S1 Chemicals and reagents
- S2 Instrumental analysis
- Table S1: Gradient elution
- Table S2: Instrument and ESI source parameters
- Figure S1: Recoveries of C_4-C_{10} PFCAs in eluate, filtrate and washing step
- Figure S2: Calibration curve of TFA

Instrumental analysis

Table S 1: Gradient elution used for HPLC-QTOF-MS measurements. Eluent A: 95/5 water/MeOH + 2 mM NH_4Ac, eluent B: 5/95 water/MeOH + 2mM NH_4Ac.

6550 QTOF-MS		
**Time (min)**	**A (%)**	**B (%)**
0.0	85	15
2.0	30	70
5.0	10	90
10.0	0	100
15.0	0	100
15.1	85	15
22.0	85	15

Table S 2: Instrument and ESI source parameters used during HPLC-QTOF-MS measurements.

**Instrument parameters**	
Gas Temp (°C)	150
Gas Flow (L/min)	16
Nebulizer pressure (psig)	35
Sheath gas temperature (°C)	380
Sheath gas flow (L/min)	12
Fragmentor voltage (V)	360
**Ion source parameter (ESI)**	
Capillary voltage (V)	3000
Nozzle voltage (V)	300

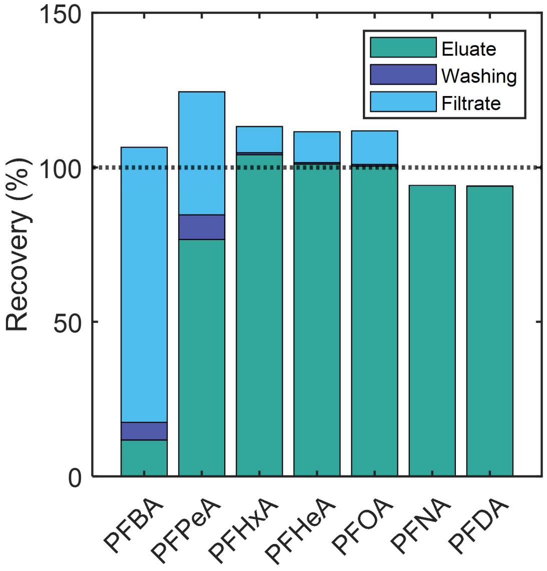

Figure S 1: Recoveries of C_4-C_{10} PFCAs in eluate, filtrate and washing step, using the described SPE method with OASIS HLB cartridges.

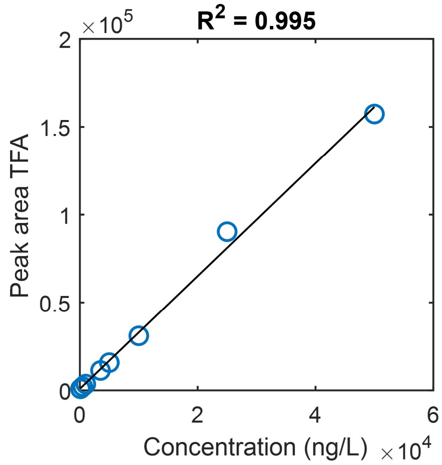

Figure S 2: Calibration curve of TFA measured with an isocratic method on a HPLC-QTOF-MS in negative ESI.

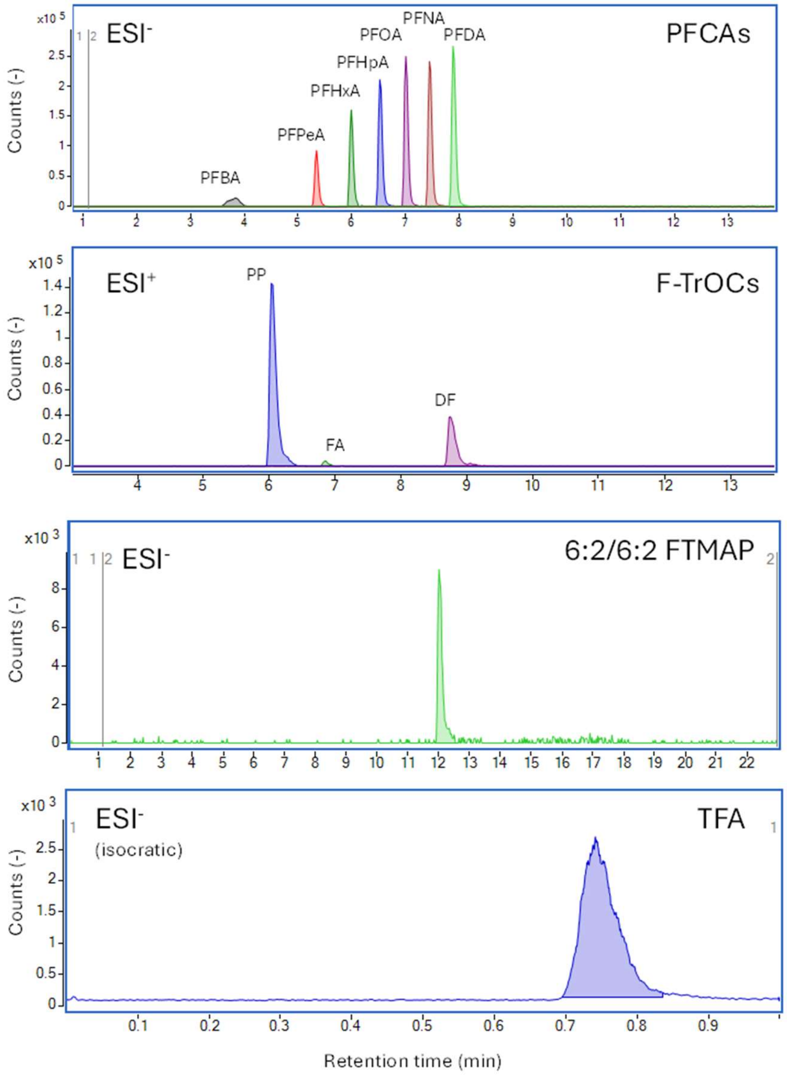

Figure S 3: Extracted ion chromatograms (EICs) of all measured compounds (PFCAs, F-TrOCs, 6:2/6:2 FTMAP, and TFA) from standards.

References

Bugsel, B., Bauer, R., Herrmann, F., Maier, M.E., Zwiener, C., 2022. LC-HRMS screening of per- and polyfluorinated alkyl substances (PFAS) in impregnated paper samples and contaminated soils. Anal Bioanal Chem 414, 1217-1225.
